# Limbic network co‐localization predicts pharmacoresistance in dysplasia‐related epilepsy

**DOI:** 10.1002/acn3.51892

**Published:** 2023-09-12

**Authors:** Nathan T. Cohen, Phat Chang, Taha Gholipour, Chima Oluigbo, L. Gilbert Vezina, Hua Xie, Anqing Zhang, William D. Gaillard

**Affiliations:** ^1^ Center for Neuroscience Research Children's National Hospital, The George Washington University School of Medicine Washington DC USA; ^2^ Division of Biostatistics and Study Methodology Children's National Research Institute Washington DC USA

## Abstract

To evaluate the role of focal cortical dysplasia co‐localization to cortical functional networks in the development of pharmacoresistance. One hundred thirty‐six focal cortical dysplasia patients with 3.0 T or 1.5 T MRI were identified from clinical databases at Children's National Hospital. Clinico‐radio‐pathologic factors and network co‐localization were determined. Using binomial logistic regression, limbic network co‐localization (odds ratio 4.164 95% confidence interval 1.02–17.08, *p* = 0.048), and focal to bilateral tonic–clonic seizures (4.82, 1.30–18.03, *p* = 0.019) predicted pharmacoresistance. These findings provide clinicians with markers to identify patients with focal cortical dysplasia‐related epilepsy at high risk of developing pharmacoresistance and should facilitate earlier epilepsy surgical evaluation.

## Introduction

Focal cortical dysplasia (FCD) is the most common etiology of surgically‐remediable, pharmacoresistant epilepsy (PRE) in childhood.^1^, ^2^, ^3^ Most children with FCDs with seizures develop PRE.^4^ Age of seizure onset, cortical lobar location, and FCD pathologic subtype are not related to development of FCD PRE.^3^ There is growing evidence for FCD‐related epilepsy as a network disorder. FCD network co‐localization may follow ontogenesis: FCDs in somatomotor and visual networks exhibit earliest age of epilepsy onset, and those in associative networks (e.g., limbic) the latest onset.^5^ Focal epilepsy may cause distinct alterations in resting state functional connectivity networks (see Royer et al. 2022^6^ for comprehensive review). The purpose of this study was to investigate whether FCD co‐localization to a specific network is associated with risk of pharmacoresistance.

## Methods

This is an IRB‐approved, retrospective cohort study at Children's National Hospital. Patients were identified via search of a radiology or surgery databases using “focal cortical dysplasia.” Patients (age 0–22 years) were included if they had 3.0 T or 1.5 T MRI‐confirmed FCD between 1/2011–1/2021 with at least 18 months follow‐up (unless diagnosis was single seizure or incidental FCD in which case any amount of follow‐up was included). MRIs were reported by board‐certified pediatric neuroradiologists with experience in pediatric epilepsy surgical evaluations (including LGV) and reviewed independently by NTC. Patients were excluded if: dual pathology (except Type IIIa^7^ (FCD with mesial temporal sclerosis)), hemimegalencephaly, or tuberous sclerosis complex. Current views are that the FCD is an in utero neuronal migration abnormality and MTS is an acquired lesion, at the earliest at end of the first year of life^8^ and likely follows seizures engendered by seizures emanating from the adjacent FCD. Direct causal association is difficult to establish and MTS may arise for other reasons not well elucidated.

PRE is defined as starting a third antiseizure medication (ASM), for failure of two adequately‐dosed, appropriately selected ASM (in line with ILAE definition of PRE^9^), or having undergone epilepsy surgery (with documented failure of two ASM but specific date of third ASM initiation was not charted). Pharmacosensitive epilepsy (PSE) is epilepsy controlled by one or two adequately‐dosed, appropriately‐selected ASMs. For analysis, any patient with epilepsy not seizure‐free at the last visit (but not yet meeting PRE criteria) was included in the PSE group. Ketogenic diet was considered an ASM. Patients with single lifetime seizure or febrile seizures (any number) were included in single seizure category. FCD lobe was defined as frontal, occipital, parietal, temporal, or other. FCD pathology was defined as Type I, Type II, Type III, or unknown.

### Image analysis

**Figure 1:**
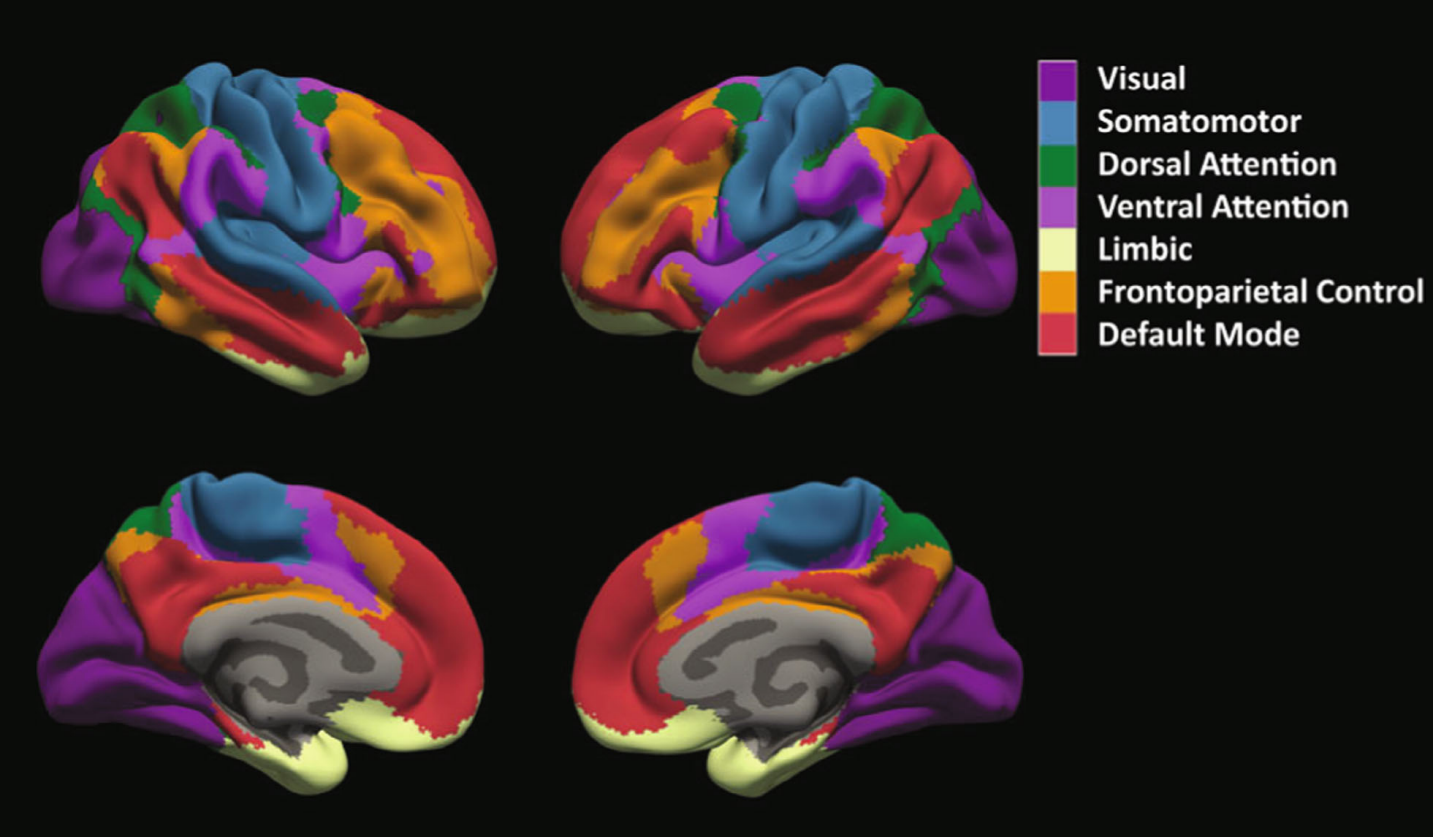
The Yeo 7‐network parcellation. This figure demonstrates the Yeo 7‐network parcellation projected onto cortical surfaces (clockwise from top left) left lateral hemisphere, right lateral hemisphere, right mesial hemisphere, left mesial hemisphere. The network projections are color‐coded as shown in the legend.

MRIs were processed using our pipeline to determine FCD co‐localization to cortical functional networks.^5^ Briefly, lesion masks were drawn (NTC) on preoperative 3 T or 1.5 T T1, or T2 images using FSLeyes software, co‐registered to the native spoiled gradient recalled acquisition MRI (SPGR) image, and warped to Montreal Neurological Institute (MNI) space. Yeo 7‐network parcellation (Fig. 1) was applied.^10^ This adult‐based parcellation is validated in the identification of pediatric functional networks as young as 3 years old.^11^ Combined mask and parcellated brain networks were warped back to native space. FCD dominant network is the Yeo network with the largest percentage lesion overlap.

### Statistical analysis

Chi‐square test was used for categorical comparisons. Outcome was binarized as PRE status (PRE or not); Chi‐square was used to evaluate FCD cortical lobar location versus PRE status; FCD dominant network versus PRE status; FCD pathologic subtype versus FCD dominant network.

Mann–Whitney U test was used to evaluate the relationship between percentage FCD network overlap versus PRE status, and focal to bilateral tonic–clonic seizures (FTBTC) or not.

Binomial logistic regression was used to determine predictors of the binary outcome of PRE or not PRE (including PSE or single seizure and excluding those with incidental FCD and no seizure history to add age of seizure onset to the regression analysis). FCD pathologic subtype could not be evaluated in this regression as non‐operated patients do not have pathology. In all tests, *p* < 0.05 was considered significant. Odds ratios were reported with 95% confidence intervals. Statistical analysis was performed using Jamovi (https://www.jamovi.org/).

Anonymized data are available by request from any qualified investigator.

## Results

One hundred thirty‐six patients (56% male) were included. Median age of seizure onset was 3.0 years (IQR 0.5–5.5y), and median follow‐up 49.5 months (IQR 25‐74mo). One hundred eighteen patients had epilepsy (87%): PRE (*n* = 84, 62%); PSE (*n* = 34, 25%). Eleven patients (8%) had a single seizure (unprovoked or febrile seizure). Seven patients (5%) had incidental FCD without history of seizure. FCD cortical lobar distribution was frontal (*n* = 62, 46%), temporal (*n* = 41, 30%), parietal (*n* = 17, 12%), occipital (*n* = 10, 7%), cingulate (*n* = 5, 4%), insula (*n* = 1, 1%). There was no effect of FCD cortical lobe on PRE (*p* = 0.313).

Seventy‐two patients had available pathology: Type I (*n* = 22), Type II (*n* = 44), Type III (*n* = 5), unknown (*n* = 1). Pathology had no effect on FCD dominant network distribution (*p* = 0.441).

### Network analyses

The overall distribution of FCD dominant network was limbic (*n* = 33, 24%), DMN (*n* = 32, 24%), somatomotor (*n* = 18, 13%), frontoparietal (*n* = 16, 12%), dorsal attention (*n* = 16, 12%), visual (*n* = 11, 8%), ventral attention (*n* = 10, 7%).

Of the 84 patients with PRE, the FCD dominant network was limbic (*n* = 27, 32%), DMN (*n* = 17, 20%), dorsal attention (*n* = 10, 12%), frontoparietal (*n* = 10, 12%), somatomotor (*n* = 8, 10%), visual (*n* = 7, 8%), ventral attention (*n* = 5, 6%) (Table 1). In univariate analysis, FCD dominant network was not associated with PRE outcome (*p* = 0.356). FCD percentage overlap with limbic network was associated with PRE outcome (*p* = 0.005). Percentage overlap with DMN but not limbic network was associated with FTBTC seizures (*p* = 0.011 and *p* = 0.546).

**Table 1:** Functional network co‐localization and pharmacoresistant epilepsy.

PRE or not PRE	Dominant network							Total
	Ventral attention	Somatomotor	Dorsal attention	Default mode	Visual	Frontoparietal	Limbic	
PRE	5	8	10	17	7	10	27	84
Not PRE	4	8	6	12	4	5	6	45
Total	9	16	16	29	11	15	33	129

This table demonstrates the distribution of functional network co‐localization and outcome of PRE or Not PRE.

#### Dominant network model

Binomial logistic regression was used to evaluate multiple predictors of the binary outcome PRE or not (Table 2). The model evaluated these predictors—age of seizure onset, hemisphere, presence of FTBTC seizures, FCD dominant network. Limbic FCD co‐localization was associated with PRE outcome (*p* = 0.003, OR 4.164 95% confidence interval (CI) 1.02–17.08, p = 0.048). Hemisphere was unrelated to PRE outcome. Age of seizure onset was negatively correlated to PRE outcome (OR 0.838, 95% confidence interval [CI] 0.76–0.93, *p* < 0.001)—younger age of onset is associated with increased risk of PRE. The presence of FTBTC seizures predicted PRE outcome (OR 4.82 95% CI 1.30–18.03, *p* = 0.019).

**Table 2:** Multivariable binomial logistic regression of predictors of pharmacoresistant epilepsy.

Predictor	Estimate	SE	*Z*	*p*	Odds ratio	95% confidence interval	
						Lower	Upper
Intercept	0.5271	0.5929	0.8891	0.374	1.694	0.530	5.415
FTBTC							
Yes – no	1.5731	0.6729	2.3379	0.019	4.822	1.290	18.027
Age seizure onset	−0.1769	0.0526	−3.3634	< .001	0.838	0.756	0.929
FCD hemisphere							
L – R	0.4654	0.4512	1.0317	0.302	1.593	0.658	3.856
Dominant network							
VentAttn – Somatomotor	0.3881	0.9225	0.4207	0.674	1.474	0.242	8.990
DorsAttn – Somatomotor	0.8480	0.8101	1.0468	0.295	2.335	0.477	11.424
Default – Somatomotor	−0.0855	0.6753	−0.1266	0.899	0.918	0.244	3.449
Visual – Somatomotor	0.0643	0.8424	0.0763	0.939	1.066	0.205	5.559
Frontoparietal – Somatomotor	0.7184	0.7799	0.9211	0.357	2.051	0.445	9.460
Limbic – Somatomotor	1.4265	0.7200	1.9812	0.048	4.164	1.015	17.076

Estimates represent the log odds of “PREornot = PRE” versus “PREornot = Not PRE”. This table shows the results of the multivariable binomial logistic regression for the outcome of pharmacoresistant epilepsy or not and predictors focal to bilateral tonic clonic seizures (FTBTC), age of seizure onset, FCD hemisphere, and dominant network (ventral attention [VentAttn], dorsal attention [DorsAttn], default mode, visual, frontoparietal, somatomotor). The model fit measures are as follows: deviance (142); Aikaike's Information Criterion (162); McFadden pseudoR^2^ (0.148). Overall model tests: Chi‐square (24.7); degrees of freedom (9); *p* = 0.003.

## Discussion

Our multivariate model finds that FCD co‐localization to the limbic network is associated with PRE. A clinical history of FTBTC seizures holds similar, independent odds as FCD limbic co‐localization in predicting PRE. Numerous limbic network functional and structural connectivity changes are related to pharmacoresistant temporal lobe epilepsy,^6^, ^12^ but no specific signature of pharmacoresistance is currently identified. Our prior data found FCD predilection for the limbic network and DMN.^13^ The present results build on these findings indicating the importance of limbic network co‐localization in FCD PRE.

There may be multiple networks or interactions that drive pharmacoresistance. FCDs have higher connectivity in anterior DMN and dorsal attention networks than controls.^14^ One possible reconciliation for these multiple network findings is that FCD‐related epilepsy may affect inter‐network hub nodes, leading to connectome alterations involved in the generation and propagation of focal seizures.^6^ Network‐based, data‐driven profiling of FCDs showed different patterns of intra‐ and inter‐network connectivity thought likely based on nodal interactions and less on structural MRI appearance; that is, morphologically discrete dysplasias were often hyperconnected and associated with larger disruptions of whole brain network topology.^15^ These connectivity differences affect surgical outcomes^16^ and can normalize after FCD removal.^17^ FCD is an excellent natural model for future connectomics analyses to elucidate the functional signatures of pharmacoresistant focal epilepsy.

There are limitations in our study. The single center design risks recruitment bias, which is minimized by using the entire hospital system's radiology database to identify all FCD patients (with and without epilepsy) including incidentally discovered lesions. Our enter is a large pediatric hospital with extensive regional primary/secondary/tertiary care capabilities managing >90% of the epilepsy population (from seizure onset) in the Washington, D.C. metropolitan region. Cortical functional network architecture may be undersampled by discrete parcellations and not account for the possibility of regions with multiple network membership. Lesion mapping does not explore connectivity alterations, however we used a network parcellation that is data‐driven and reproducible across adult and pediatric datasets with high reliability.^18^ The PSE group may include patients who later develop PRE; this was a choice to increase the specificity of the PRE group. Future prospective studies are needed to characterize better the non‐epilepsy population, as well as more fully evaluate surgical outcomes using a network‐based approach.

These findings provide useful imaging and clinical markers that may identify children with FCD‐related epilepsy for timely consideration of epilepsy surgery. Future investigations should explore the specific network connectivity alterations underlying pharmacoresistance including subcortical networks.

## Author Contributions

**Nathan T. Cohen:** Conception and design of study, acquisition and analysis of data, drafting a significant portion of the manuscript or figures. **Phat Chang:** Acquisition and analysis of data, drafting a significant portion of the manuscript or figures. **Taha Gholipour:** Acquisition and analysis of data, drafting a significant portion of the manuscript or figures. **Chima Oluigbo:** Acquisition and analysis of data, drafting a significant portion of the manuscript or figures. **L. Gilbert Vezina:** Acquisition and analysis of data, drafting a significant portion of the manuscript or figures. **Hua Xie:** Acquisition and analysis of data, drafting a significant portion of the manuscript or figures. **Anqing Zhang:** Conception and design of study, acquisition and analysis of data, drafting a significant portion of the manuscript or figures. **William D. Gaillard:** Conception and design of study, acquisition and analysis of data, drafting a significant portion of the manuscript or figures.

## Conflict of Interest

The authors have no relevant conflicts of interest.
